# Case Report: The application of amplatzer vascular plug to repair aortic dissection intimal tears and false lumen

**DOI:** 10.3389/fcvm.2023.1337430

**Published:** 2024-01-08

**Authors:** Ruihua Li, Yang Liu, Jianjun Jiang

**Affiliations:** Department of General Surgery, Vascular Surgery, Qilu Hospital of Shandong University, Jinan, Shandong, China

**Keywords:** endovascular treatment, embolization technique, amplatzer vascular plug, coils, aortic dissection

## Abstract

In recent years, significant advancements have been made in endovascular therapy for aortic dissection, resulting in the development of various treatment methods. Nevertheless, there is a contentious discussion regarding the suitability of different treatment methods for addressing the unique features of individual lesions. Specifically, the matter of whether the presence of small intimal tears or multiple distal tears requires extensive aortic coverage with graft stents, which may block the blood supply to important organs, intercostal arteries, or lumbar arteries, remains unresolved. Further research is necessary to determine the need for complex fenestrated or branched endovascular aortic repair (F/B-EVAR) in the management of chronic post-dissection aneurysms involving visceral arteries. Our report highlights the successful endovascular repair of three patients with aortic dissection, encompassing one Stanford A type and two Stanford B types. Utilizing the Amplatzer Vascular Plug (AVP) alone or in combination with the coil embolization technique, we achieved favorable clinical outcomes. This article aims to provide valuable insights and new perspectives on the tailored management of aortic dissection by conducting a comprehensive examination of the subtle differences in the treatment processes and techniques applied to these three patients.

## Introduction

Aortic dissection is a severe cardiovascular emergency (1), occurring at a rate of 2–3 cases per 100,000 individuals in China, leading to a mortality rate of 7.3% (2). Guidelines recommend various aortic replacement surgical techniques for Stanford Type A, while Type B is treated with endovascular aortic repair (EVAR) (3, 4). Advancements in technology prompt exploration of endovascular intervention for Type A. However, both open and endovascular surgeries involve intricate procedures with significant mortality rates (5). Hence, embolization is a technique that medical professionals carefully consider for treating commonly occurring intimal tears or stable chronic aortic dissection.

**Figure 1:**
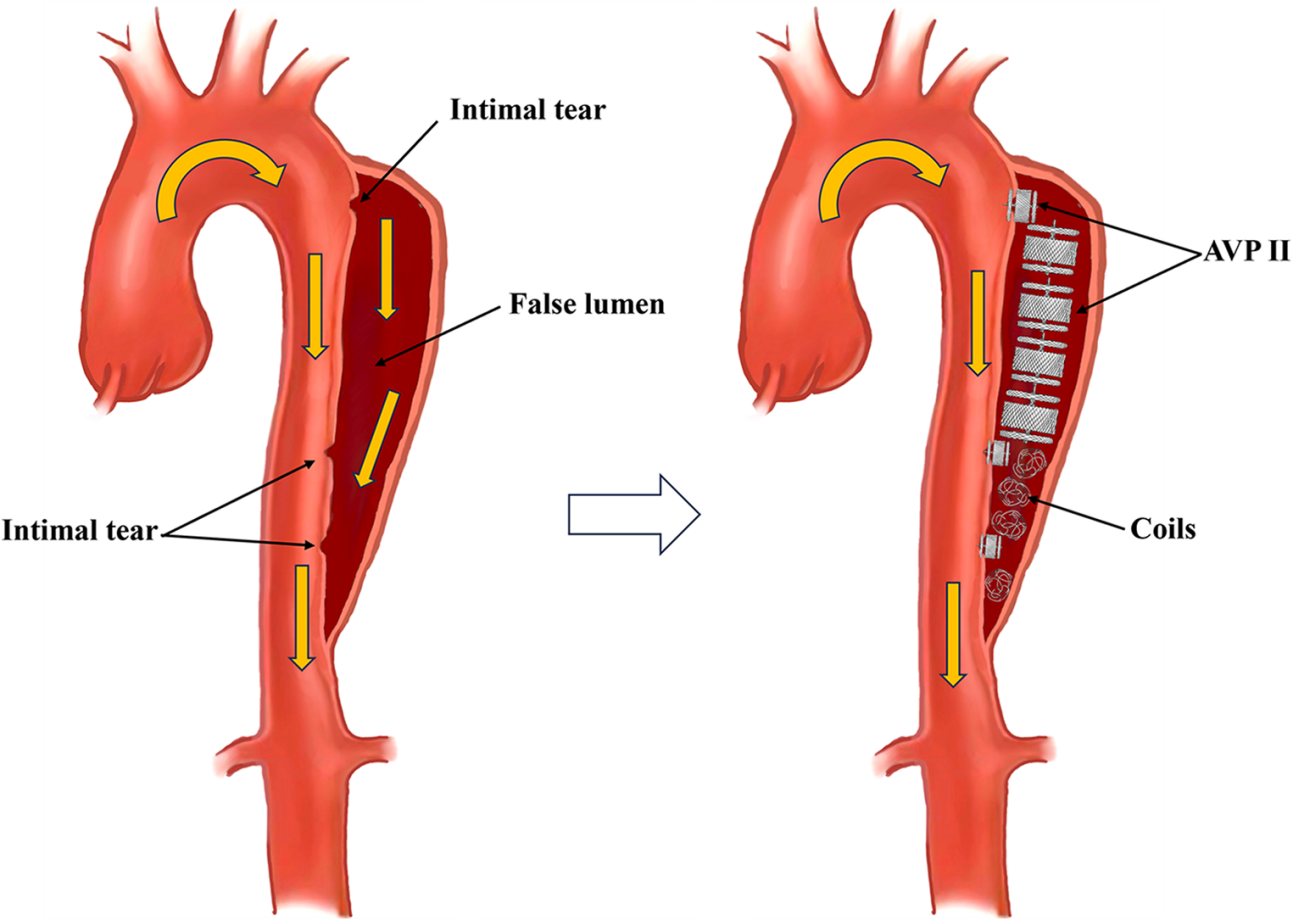
Aortic dissection treated using AVPs and coils.

The Amplatzer Vascular Plug (AVP), a vascular occlusion device, is designed to optimize embolization in various endovascular surgery applications (6, 7). Available in four models, each tailored to different vascular anatomy and clinical scenarios, AVP's application for the direct occlusion of aortic dissection entry tears has limited study, and evidence supporting its safety and efficacy is insufficient. Our center selectively treated three patients with Stanford A and Stanford B type aortic dissection, using AVP II (Abbott, Minnesota, USA) to close intimal tears or combining it with coils for false lumen embolization (Table 1). Our initial idea was to use embolization techniques to reconstruct the aneurysm neck, addressing post-dissection aortic aneurysms involving visceral arteries. This approach aimed to avoid complex surgeries for preserving visceral arteries and to create conditions for standard EVAR procedures (Figure 1). To our delight, the results of the first surgery exceeded our expectations, with both patients showing complete thrombosis of the false lumen, eliminating the need for subsequent procedures in the true lumen. In this paper, we delve into the analysis of the treatment process and technical intricacies, aiming to provide valuable experiences and fresh perspectives for the surgical management of aortic dissection.

**Table 1:** Demographics of 3 patients with chronic dissection treated with amplatzer vascular plug II.

		Patient 1	Patient 2	Patient 3
Age (years)		51	62	38
Sex		Female	Male	Male
Stanford type (A/B)		A	B	B
Previous aortic surgery		TEVAR for aortic dissection 2 years ago	F/B-EVAR for aortic arch and thoracic aorta 9 months ago	F/B-EVAR for thoracic and abdominal aorta 2 years ago
Tears position		ascending aorta; infrarenal abdominal aorta; right renal artery	abdominal aorta near the celiac artery; both iliac arteries	infrarenal abdominal aorta; right iliac artery
Surgical procedure	Step 1	Under general or general anesthesia, femoral artery access was performed.		
	Step 2	Intraoperative angiography is used to identify the number, location, and size of tears, determine the extent of involvement of the false lumen, and evaluate the blood supply to visceral branches.		
	Step 3	4 mm × 7 mm AVP II blocked ascending aorta tear	Coils embolize the narrowed false lumen	Procedure 1: 16 mm, 20 mm, and 22 mm AVP II were used to embolize the false lumen
	Step 4	7 × 25 mm Viabahn stent blocked right renal artery tear	14 mm, 18 mm, 20 mm, 22 mm AVP II were used to embolize the false lumen	Procedure 2: 8 mm × 6 mm AVP II blocked abdominal aorta tear
	Step 5	10 × 7 mm AVP Ⅱ blocked infrarenal abdominal aorta tear	Coils embolize the distal false lumen	Procedure 2: 20 × 16 mm AVP II and coils were used to embolize the false lumen
	Step 6			Procedure 2: 13 mm × 50 mm Viabahn stent and 10 mm × 80 mm Fluency stent in right iliac artery TL
Follow-up (months)		6	3	6
Outcome		Successful	Successful	Successful

TEVAR, thoracic endovascular aortic repair; F/B-EVAR, fenestrated or branched endovascular aortic repair; AVP II, amplatzer vascular plug II; TL, true lumen.

## Case report

Case 1: A 51-year-old female underwent thoracic endovascular aortic repair (TEVAR) for aortic dissection 2 years ago. The patient was readmitted because of persistent chest discomfort. Computed tomography angiography (CTA) revealed multiple tears at the origin of the ascending aorta and around the renal artery, with post-dissection aneurysm formation (Supplementary Figures S1A,C).

**Figure 2:**
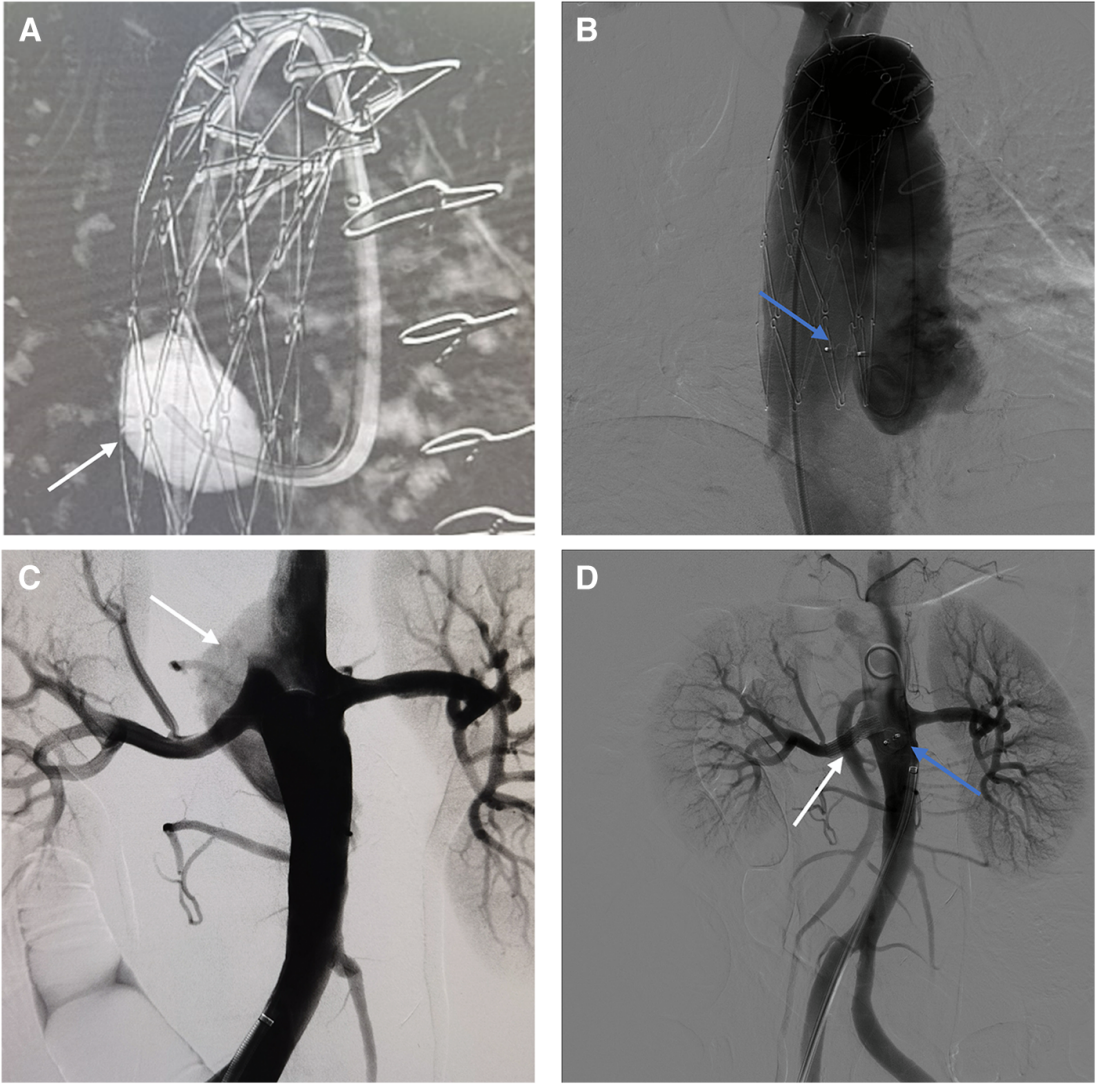
(**A**) Intraoperative imaging of aneurysm cavity. (white arrow) (**B**) Use AVP to embolize the tear of ascending aortic. (blue arrow) (**C**) The false lumen of the abdominal aorta. (white arrow) (**D**) Final intraoperative angiography showed the AVP (blue arrow) and the stent (white arrow).

Under general anesthesia, a 6 F catheter was inserted through the right femoral artery to perform false lumen angiography in the ascending aorta (Figure 2A). A 4 mm × 7 mm AVP II was placed in the tear, with two parts in the false lumen and one in the true lumen. Aortography confirmed proper placement of AVP and absence of false lumen opacification (Figure 2B). Abdominal aortography used to find tear near renal artery (Figure 2C). A 7 × 25 mm Viabahn stent (W. L. Gore & Associates, Flagstaff, AZ, USA) was placed in the right renal artery to block the tear at the opening of the renal artery. Then, using a 7F guide catheter, we inserted an AVP II measuring 10 × 7 mm into the tear in the abdominal aorta. Aortography confirmed proper AVP placement with smooth blood flow in the right renal artery stent, and no false lumen opacification (Figure 2D). After 6 months, the follow-up CTA showed complete thrombosis in the false lumen of both the ascending and abdominal aorta without endoleaks. The AVP position remained stable. The abdominal aorta diameter decreased from 51 to 38 mm, while the true lumen diameter increased from 20 to 27 mm (Supplementary Figures S1B,D).

**Figure 3:**
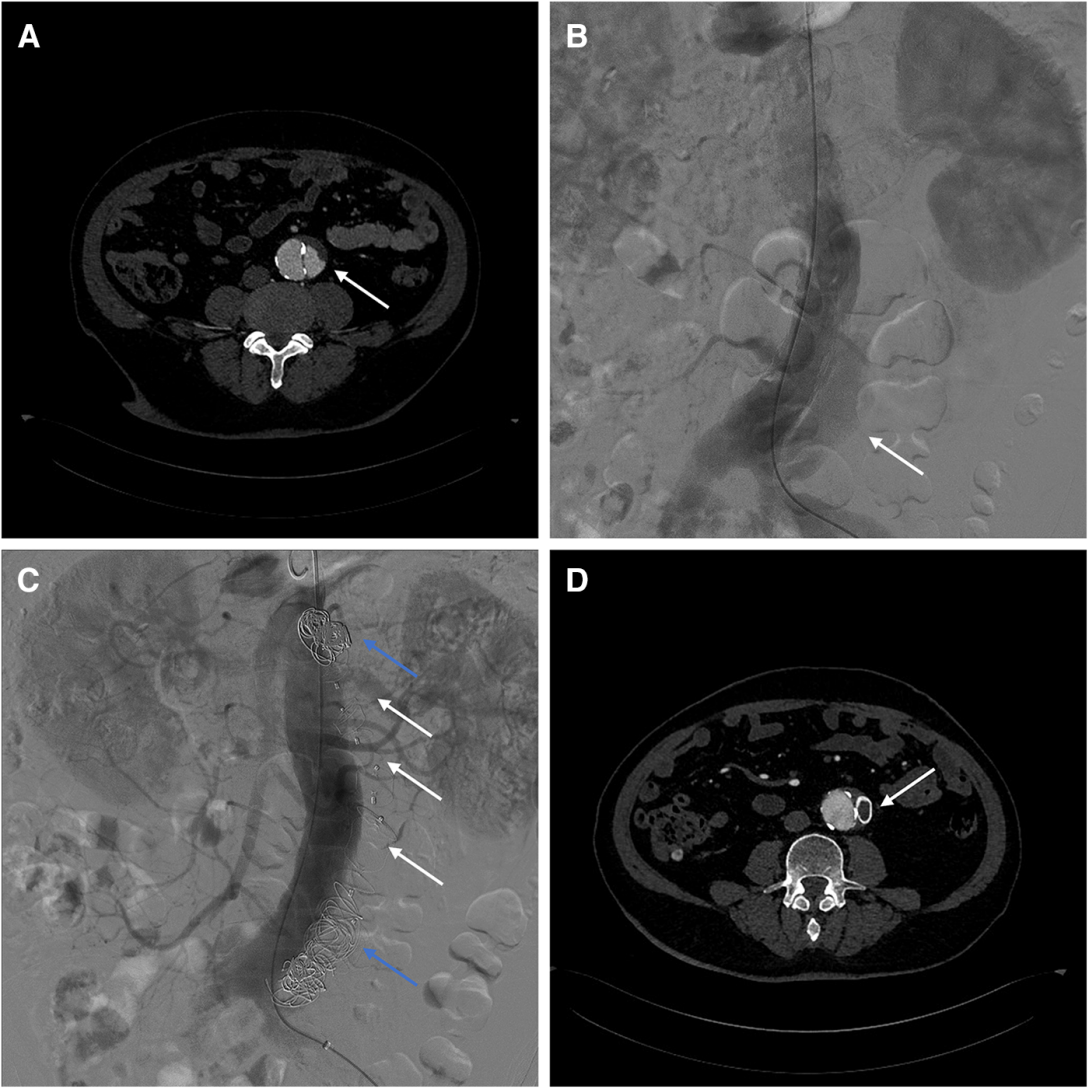
(**A**) Preoperative CTA showed blood flow in false lumen of aorta. (**B**) Intraoperative angiography showed the false lumen opacification in abdominal aorta. (**C**) Angiography showed no opacification of the false lumen after AVPs (white arrow) and coils (blue arrow) placement. (**D**) 3-month post-surgery CTA shows AVP in abdominal aorta.

Case 2: A 62-year-old male underwent fenestrated or branched endovascular aortic repair (F/B-EVAR) for aortic arch and thoracic aorta 9 months ago for type B aortic dissection. Post-surgery CTA revealed a 57 mm Post-dissection abdominal aortic aneurysm extending to both iliac arteries. Multiple tears were located at the level of the celiac trunk and bilateral iliac arteries (Figure 3A).

To avoid complex endovascular techniques, the goal is to reconstruct the aneurysm's neck as a healthy anchoring site before implementing standard EVAR in the second phase. Under local anesthesia, the false lumen is accessed through an intimal tear in the left iliac artery (Figure 3B). Due to limited space, several Interlock mechanically detachable coils (Boston Scientific, Natick, MA, USA) were inserted at the level of the celiac trunk artery. After that, AVP II with diameters of 14 mm, 18 mm, 20 mm, and 22 mm were released sequentially in the false lumen. Finally, multiple coils (COOK, Bloomington, IN, USA) were placed at the distal false lumen to aid with embolization. Angiography confirmed no false lumen opacification (Figure 3C). Postoperative CTA examination after 3 months showed complete thrombosis of the false lumen in the abdominal aorta. The diameter of the abdominal aorta decreased from 57 mm to 47 mm, while the diameter of the true lumen increased from 22 mm to 37 mm (Figure 3D). Therefore, a second-stage EVAR surgery is not necessary.

Case 3: A 38-year-old male underwent F/B-EVAR for type B aortic dissection 2 years ago. Postoperative CTA follow-up: The patient has a post-dissection abdominal aortic aneurysm, with a maximum diameter of approximately 52 mm. The intimal tears are located at the level of the infrarenal abdominal aorta and the right common iliac artery respectively. All visceral arteries originate from the true lumen (Supplementary Figure S2A).

**Figure 4:**
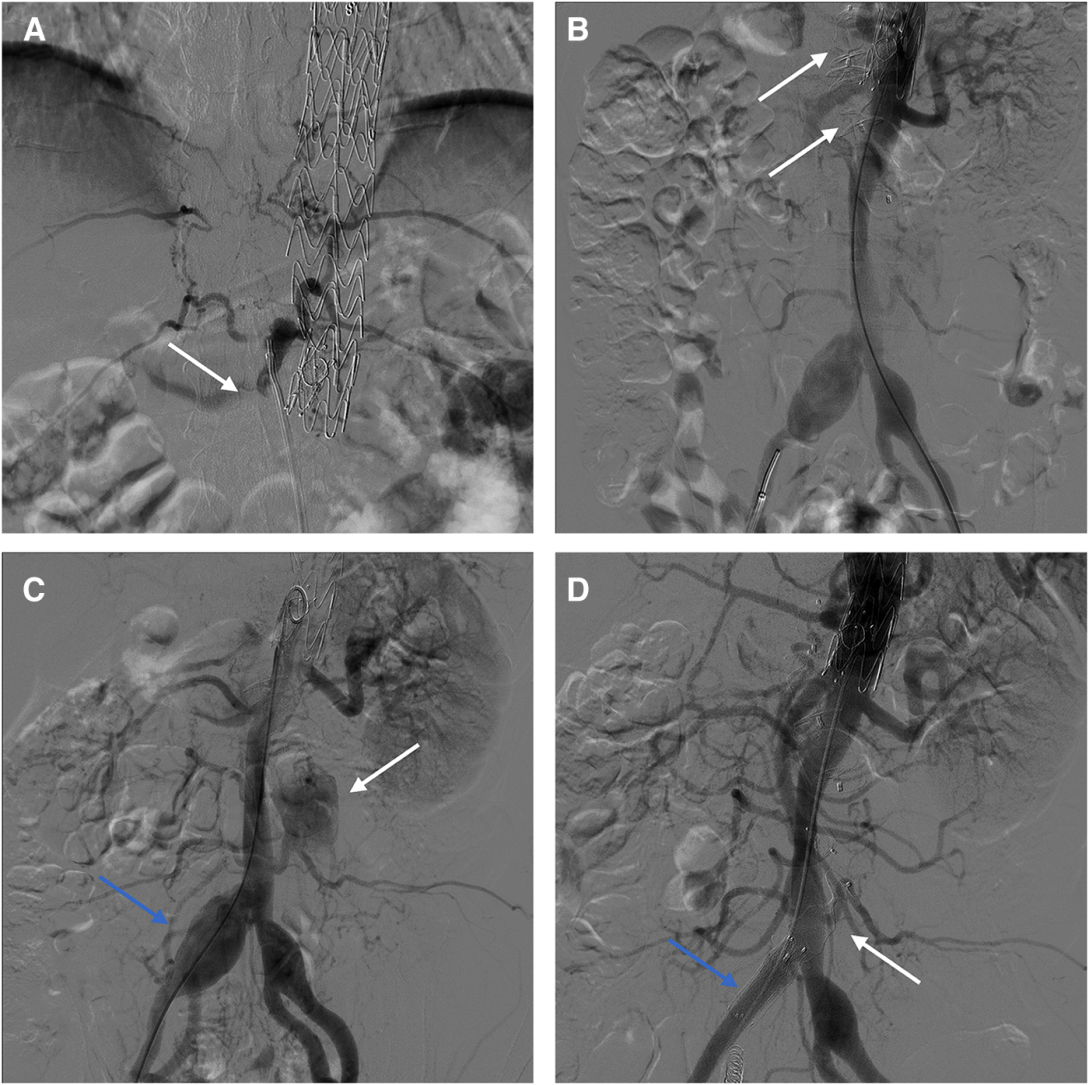
(**A**) Intraoperative angiography showed abdominal aortic false lumen opacification. (**B**) AVPs were inserted into the false lumen. (**C**) Opacification of false lumen in infrarenal aorta (white arrow); Aneurysmal dilatation of right iliac artery (blue arrow). (**D**) Final intraoperative angiography showed the AVP (white arrow) and the stent (blue arrow).

Procedure 1: Under local anesthesia, the false lumen was accessed via a tear in the right iliac artery (Figure 4A). Subsequently, AVP II with diameters of 16 mm, 20 mm, and 22 mm were placed to embolize the false lumen (Figure 4B). Intraoperatively, angiography revealed that several lumbar arteries arose from the false lumen. Since the therapeutic goal had been attained, the procedure was terminated to prevent acute spinal cord ischemia. The second phase of the surgery is scheduled to take place in a few months. Follow-up CTA revealed persistent blood flow in the mid to distal false lumen of the abdominal aorta, with distal narrowing, indicating the possibility of re-embolization therapy (Supplementary Figures S2B,C). Procedure 2: Under local anesthesia, right femoral artery access. Angiography showed post-dissection aortic aneurysm formation, and the tear was located in the infrarenal abdominal aorta and right iliac artery (Figure 4C). The 8F guiding catheter passed through iliac artery tear into false lumen, then entered true lumen via abdominal aorta tear. Placed 8 mm × 6 mm AVP II at tear site. A 20 × 16 mm AVP II was inserted into the stenosed false lumen, and Interlock mechanically detachable coils (Boston Scientific, Natick, MA, USA) was used to embolize the right internal iliac artery. Subsequently, a 13 mm × 50 mm Viabahn stent (W. L. Gore & Associates, Flagstaff, AZ, USA) and a 10 mm ×80 mm Fluency stent (Bard, Temple, AZ, USA) were placed in the right iliac artery to block the tear. Intraoperative angiography reveals patent visceral arteries and no false lumen opacification (Figure 4D). The 6-month postoperative CTA examination showed stable positions of the AVP and stent graft, with complete thrombosis in the false lumen. The abdominal aorta diameter decreased from 52 mm to 47 mm, while the true lumen increased from 24 mm to 28 mm (Supplementary Figure S2D).

## Discussion

After TEVAR, the persistence of distal tears impedes the complete closure of the false lumen, leading to inadequate thrombus formation and facilitating the advancement of distal dissection. This becomes a primary reason for secondary surgical treatment and sparks debates on whether to simultaneously seal the distal tear during TEVAR (8, 9). Currently, various techniques exist in the field, such as F/B-EVAR, chimney technique, embedded branch stent, provisional extension to induce complete attachment (PETTICOAT) technique, stent-assisted balloon-induced intimal disruption and relamination in aortic dissection repair (STABILISE) technique, Knickerbocker, etc (10, 11).

Although significant progress has been made in endovascular treatment of aortic dissection, there remain numerous unresolved issues including etiology, pathophysiology, anatomical changes, prognosis, as well as treatment strategies, techniques, and various materials and tools. Currently, clinical research is primarily focused on the improvement of treatment modalities and materials. Due to factors such as aortic compliance and compatibility, the use of covered stents in treatment leads to a relatively high rate of long-term reintervention. For anatomically complex aortic dissections, there has been an emergence of numerous surgical approaches in recent years. The selection of appropriate individualized treatment should be based on a deep understanding of the disease, extensive clinical experience, and advanced technical skills. Currently, commonly chosen techniques for selective patients include chimney technique and F/B-EVAR technique (12, 13). While these methods can achieve the desired therapeutic effects, they also come with a high rate of re-intervention (14, 15) and significant economic costs. Therefore, it is crucial to simplify treatment strategies and procedures based on patient benefit.

At present, there have been only a few case reports that have described the application of AVP for selectively sealing tears and filling false lumens. Compared to other surgical methods, the use of AVP closure for tears has advantages such as less trauma and lower economic costs. Moreover, the most important aspect is that this technique is relatively simple in terms of operation. In 2012, Yeom et al. (16) published the first documented case of employing AVP Ⅱ for the occlusion of a distal tear in chronic type B aortic dissection. Subsequent studies have demonstrated a high success rate of AVP occlusion in sealing the tear, as well as favorable long-term aortic remodeling (17–20). AVP is a nickel-titanium mesh occlusion device with multiple layers. It is designed to be precise in positioning, controllable in release, and retrievable. AVP selectively blocks the tears, thereby preventing extensive ineffective coverage of the graft within the true lumen. This significantly lowers the risk of spinal cord ischemia and eliminates the possibility of complications related to the stent.

Using AVP to occlude the tears in combination with false lumen embolization effectively solves the problem of incomplete tears occlusion and small hidden endoleaks. These two methods can also be applied alone. It's worth exploring the use of false lumen embolization to treat post-dissection aortic aneurysm that involve the visceral arteries. This technique can effectively rebuild a healthy anchor zone, which means we can avoid complicated surgeries and even obviating the need for further EVAR.

Based on the concept of simplifying surgery, we employed embolization techniques for selective treatment in three patients with aortic dissection, including one case of Stanford type A, achieving better-than-expected outcomes. The technique procedure is relatively simple, and there's no need for excessive placement of stents within the true lumen.

The AVP occlusion of the intimal tears is applicable for the treatment of select patients with type A and type B aortic dissection. However, strict patient selection is required due to limitations in materials and intimal stability. Firstly, the diameter of the AVP should correspond to the size of the intimal tear, so it's important to choose a tear that is both more regular in shape and smaller than the diameter of the AVP. Furthermore, in the acute phase, the endarterium is fragile and morphologically unstable, with the size of the tears prone to change. There is a potential risk of device displacement and endarterium tearing when AVP is used, making thorough preoperative assessment extremely important. The chronic phase of aortic dissection exhibits a stable intimal flap, which makes it suitable for embolization technique treatment. However, there are also issues such as a relatively large diameter of the entry tears. Therefore, precise measurements should be taken before surgery to select an appropriate AVP. Thirdly, in the area of visceral arteries with true and false lumens blood supply, embolization of the false lumen should be combined with the treatment of graft stents for the visceral arteries to avoid organ ischemia. In the occlusion of AVP with intimal tears located at the opening of cervical artery and visceral artery, it is crucial to exercise caution in order to prevent stenosis or unintentional closure of the arterial opening. Additionally, the use of embolization techniques alone is not suitable for ruptured or impending rupture of aortic dissection.

## Conclusion

Simplification of endovascular treatment is one of the important goals in the design of procedures for complicated aortic dissections. The selective application of embolization techniques can yield significant benefits in cases of aortic dissection or post-dissection aortic aneurysms that involve visceral arteries. Although embolization technology is not a recent technique, with continuous improvements and innovations in materials, its application will become more and more widespread. In the future, the development of specialized embolic devices for complex morphologies of aortic dissection will have significant practical value.

## Data Availability

The raw data supporting the conclusions of this article will be made available by the authors, without undue reservation.
