# Supplementary figures and images for: Case Report: The application of amplatzer vascular plug to repair aortic dissection intimal tears and false lumen
Source: Front Cardiovasc Med. 2024 Jan 8;10:1337430. doi: 10.3389/fcvm.2023.1337430 (PMC10801193; doi:10.3389/fcvm.2023.1337430)

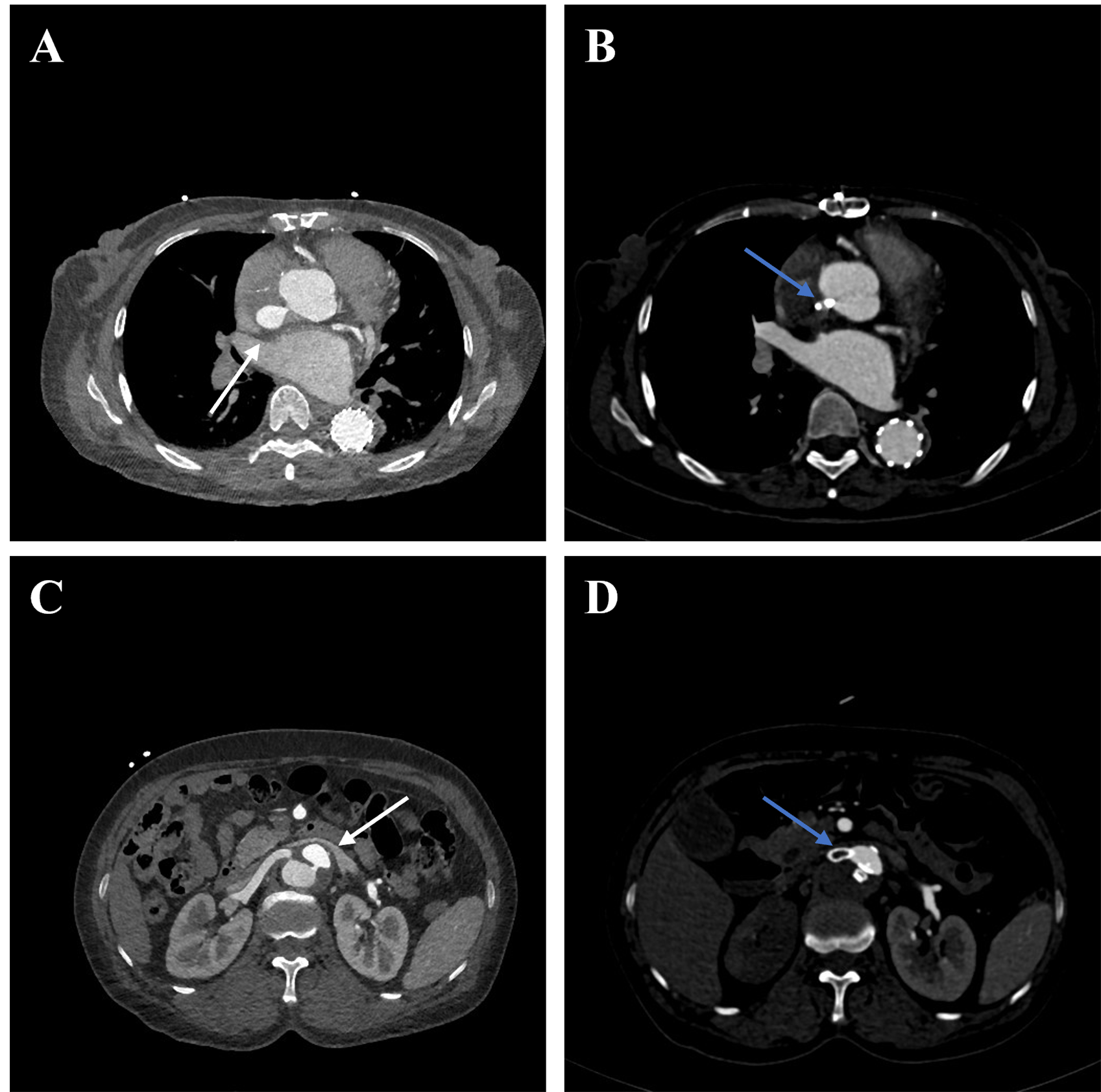

Supplement: Supplementary file 1 [file Image1.tif]

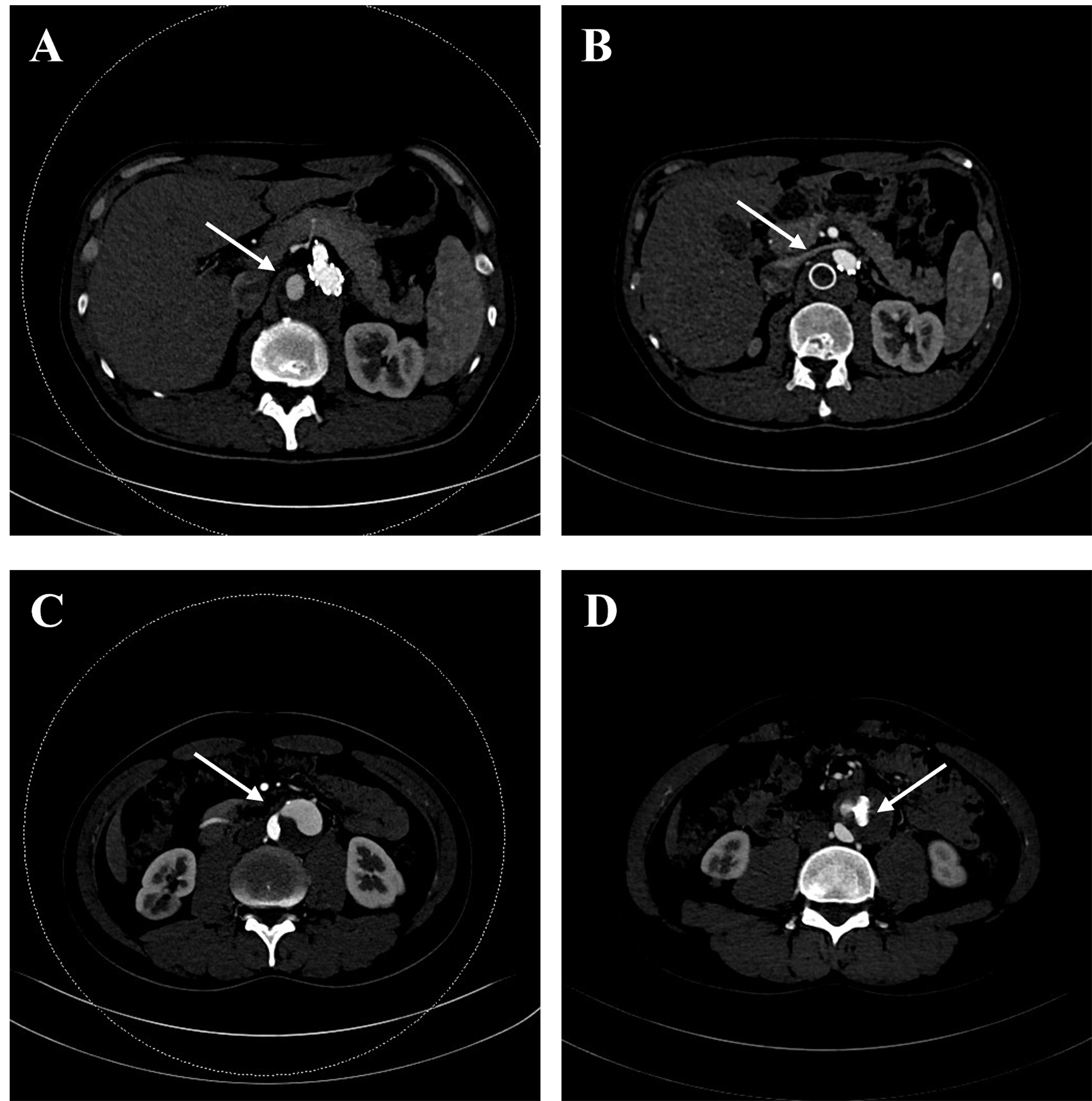

Supplement: Supplementary file 2 [file Image2.tif]
